# Supplementary material for: Pyrazinamide resistance in rifampicin discordant tuberculosis
Source: PLoS One. 2022 Sep 21;17(9):e0274688. doi: 10.1371/journal.pone.0274688 (PMC9491533; doi:10.1371/journal.pone.0274688)
Supplement: Attachment — Submitted filename: Response to reviewers comments_Plos One 2022.docx [file pone.0274688.s001.docx]

**Responses to reviewers' comments**

Reviewer #1: GENERAL

Highly relevant contribution to the field of MDRTB. Recent guidelines on the treatment of MDRTB (WHO, 2020) recommended that pyrazinamide only be considered for inclusion in drug regimens if susceptibility to the drug has been shown. Pyrazinamide is a highly effective anti-TB agent and its use would be preferred. The hypothesis in this paper is that isolates resistant to rifampicin by genotypic assay but susceptible to the drug by phenotypic assay (discordant) have a high probability for susceptibility to pyrazinamide (and also to isoniazid). Therefore, the baseline rifampicin profile has major implication for selecting an appropriate treatment regimen that also includes pyrazinamide, especially where DST for the latter agent is not available. It is, therefore, useful to define categories of MDRTB patients where pyrazinamide resistance is likely to be low and its use might be recommended as standard of care.

MAJOR COMMENT

The study investigated pyrazinamide resistance in 80 isolates classified as discordant on rifampicin gDST-pDST results. In these isolates, the pDST of rifampicin was determined using 7H10 medium and a rifampicin critical concentration of 1 ug/ml. In a 2021 technical report on critical concentrations for drug susceptibility testing of isoniazid and the rifamycins (rifampicin, rifabutin and rifapentine), the World Health Organization recommended lowering the CC of rifampicin in 7H10 and MGIT grown cultures to 0.5 ug/ml.

The authors need to elaborate on the implications of this change, if applied to their dataset, on the rationale for the study as stated in the last paragraph of the Introduction. How many of the 80 isolates would still be considered discordant if the revised CC is applied? Do the conclusions for the study hold for isolates regarded as discordant under the revised breakpoint, i.e would the probabilities for association with isoniazid and/or pyrazinamide susceptibility remain unaltered? Are there any high-confidence rpoB mutations in discordant isolates by the revised definition that would predict pyrazinamide susceptibility?

The above additional perspective is to be provided as an expansion of the existing narrative, not necessarily as a replacement of the study results and conclusions as presented.

**Response:** Thank you for this comment. The WHO lowering of rifampicin critical concentration (CC) actually strengthens our findings of low pyrazinamide prevalence in rifampicin discordant TB. 45% of the discordant isolates remain discordant after the revision of the CC. Of the six pyrazinamide resistant isolates, only one occurred within this group of isolates, the other five are amongst those who would be considered rifampicin resistant (Rifampicin MIC of >0.5 µg/mL. This means pyrazinamide resistance is even lower in rifampicin discordant TB than originally thought. We have included this information in the description of our *M. tuberculosis* isolates under the methods section as well as the results section. Additionally, we have expanded our discussion to include the effect of the change on our findings. The specific rifampicin mutations were not helpful in predicting pyrazinamide resistance, probably due to small number of PZA resistant isolates.

MINOR COMMENT

The prevalence of pyrazinamide resistance in MDRTB isolates in South Africa is high, as emphasised by the authors. Reference 24 (Ismail, et al. 2018) is quoted in the context of prevalence of rpoB mutations in a national survey. A general perspective on the magnitude of pyrazinamide resistance would be useful in stating the importance of the current work. Does the same reference provide an estimate of the level of pyrazinamide resistance? By comparison, the 2008 paper by Mphahlele, et al., DOI: 10.1128/JCM.00973-08, would be useful, also for insight into the genomics of MDRTB isolates at the time.

**Response:** We have also expanded our discussion of pyrazinamide resistance to include this information (paragraph 3 on discussion). Although pyrazinamide resistance is high among drug resistant TB isolates in South Africa, it is much lower in drug susceptible TB. In Ismail’s paper, the overall pyrazinamide resistance was only 3.7%.
